# Diarrheagenic *Escherichia coli* Pathotypes From Children Younger Than 5 Years in Kano State, Nigeria

**DOI:** 10.3389/fpubh.2019.00348

**Published:** 2019-11-27

**Authors:** Habeeb Kayode Saka, Nasir Tukur Dabo, Bashir Muhammad, Silvia García-Soto, Maria Ugarte-Ruiz, Julio Alvarez

**Affiliations:** ^1^Nigerian Stored Products Research Institute, Kano, Nigeria; ^2^Department of Microbiology, Bayero University, Kano, Nigeria; ^3^Department of Biological Sciences, Bayero University, Kano, Nigeria; ^4^VISAVET Animal Health Surveillance Centre, Universidad Complutense, Madrid, Spain; ^5^Departamento de Sanidad Animal, Facultad de Veterinaria, Universidad Complutense, Madrid, Spain

**Keywords:** diarrhegenic *Escherichia coli*, virulence factors, pathotypes, resistance, Kano state

## Abstract

Diarrheagenic *Escherichia coli* (DEC) is one of the leading causes of gastrointestinal disorders worldwide and an important public health challenge. DEC infection is often underdiagnosed during routine microbiological analysis, especially in resource constrained settings; the use of molecular tests could however help to determine the distribution of DEC and its clinical significance. Here, a study to assess the prevalence of DEC in clinical samples from patients <5 years attending three hospitals in Kano state, Nigeria, was carried out. Samples from 400 patients and 50 controls were collected and screened for *E. coli*. Compatible colonies from 248 individuals (215 patients and 33 controls) were characterized using biochemical test, a set of real-time PCRs for detection of nine virulence factors (VF: *eae, bfp*A, *elt, est, stx*1, *stx*2, *ehx*A, *agg*R, and *inv*A) associated with five DEC pathotypes (EPEC, ETEC, EHEC, EAEC, and EIEC) and antimicrobial susceptibility tests. One or more VFs typical of specific pathotypes were detected in 73.8% (183/248) of the isolates, with those associated with EAEC (36.3%), ETEC (17.3%), and EPEC (6.0%) being the most common, although proportion of specific pathotypes differed between hospitals. *est* was the only VF detected in a significantly higher proportion in cases compared to controls (*P* = 0.034). Up to 86.9% of DEC were resistant to at least one class of antibiotics, with trimethoprim-sulfamethoxazole being the least effective drug (77.6% resistance). Our results demonstrate the widespread circulation of different DEC pathotypes that were highly resistant to trimethoprim-sulfamethoxazole among children in Kano state, and highlight the need of characterizing the causative agents in cases of gastrointestinal disorders.

## Introduction

Enteric infections and diarrheal diseases (EIDD) constitute a pervasive health burden throughout the world (1–3), closely associated with poor water supply, sanitation and hygiene status, which are common in developing countries (1). EIDD affect a large number of people globally and constitute a leading cause of morbidity and mortality in developing countries, with higher rates among children and aged individuals (4). Diarrhea is characterized by stools of decreased consistency and increased volume due to imbalance of secretion and absorption of water and salts in the intestine (5). It is a major source of malnutrition and life threatening diseases which may be fatal (6, 7).

Diarrhea can be caused by a wide range of microbial agents including viruses, bacteria, and parasites (8). Among the bacterial agents, *Escherichia coli* is one of the most common causes of diarrhea. *E. coli* is a Gram-negative, oxidase-negative, rod-shaped bacterium from the family *Enterobacteriaceae* (9) that is encountered as a normal inhabitant of human and other mammalian intestine (10). It colonizes the gastrointestinal tract of new-born infants within few hours after birth (11) and is readily isolated from fecal samples. However, several well-adapted *E. coli* clones have acquired specific virulence factors (VFs) that augment their ability to cause a broad range of diseases (12). Diarrheagenic *Escherichia coli* (DEC) is responsible for about 30–40% of acute diarrhea episodes in children <5 years in developing countries (13), and is an important cause of both sporadic cases and diarrheal outbreaks all over the world (9). The VFs are encoded on mobile genetic element that can be inserted into new strains, potentially creating new combinations of VFs (10). The most successful of these combinations are used to define *E. coli* pathotypes that are capable of causing disease in healthy individuals (4, 10, 11). The major DEC pathotypes (pathogenic variants of *E. coli*) that cause high morbidity and mortality worldwide (9) are: enterotoxigenic *E. coli* (ETEC), enteroinvasive *E. coli* (EIEC), enteroaggregative *E. coli* (EAEC), diffusely adherent *E. coli* (DAEC) enteropathogenic *E. coli* (EPEC), and enterohemorrhagic *E. coli* (EHEC) (9). However, *E. coli* strains carrying certain VFs have been also recovered from healthy human controls, thus demonstrating that the presence of certain VFs does not necessarily imply their involvement in clinical disease (14).

*E. coli* is frequently isolated from diarrheic children in Kano, Nigeria, but the genetic background, including the presence of VFs, is not routinely evaluated, and hence the proportion of diarrhea attributable to DEC remains undefined. Because of this, there is a paucity of information on the importance of DEC and of specific DEC pathotypes as causative agents of diarrhea in Nigeria and Kano in particular. This study investigated the occurrence and frequency of DEC as a cause of infectious diarrhea in children younger than 5 years in Kano, along with their antibiotic susceptibility pattern, to provide baseline data on the circulating DEC pathotypes in Kano and on their clinical significance.

## Materials and Methods

This study was approved by Kano State Ministry of Health (Ref: MOH/Off/797/T.I/186) prior to commencement. The ethics sub-committee of the ministry operational research advisory committee approved the use of both informed verbal assent and written consents from children with literate parent and the use of verbal informed consent and willingness to provide sociodemographic information instead of written informed consent when participant parents were illiterate. Rectal swab specimens were obtained from 400 diarrheic children younger than 5 years attending three Hospitals in the state: Murtala Muhammad Specialist Hospital (MMSH) Kano, Wudil General Hospital (WGH), Wudil and Bichi General Hospital (BGH), Bichi presenting with diarrhea younger than 5 years, with/without vomiting and had not commenced antibiotics. Children without diarrhea, older than 5 years, children of non-consenting parents and diarrheic children that had received any antibiotic treatment were excluded from the study. Social demographic and clinical characteristics from sampled individuals were obtained using a standardized survey. In addition, 50 children without diarrhea in the last 21 days attending MMSH for causes other than intestinal problems with/without receiving antibiotic treatment were recruited as a control group. All specimens for this study were collected between April 2017 and November 2017. Additional information on the sample collection process is described elsewhere (15).

Information on specimen collection and transport was previously described in Dabo et al. (15). Briefly, specimens were collected from the subjects using sterile swab sticks containing Carry-blair medium (*Micropoint Diagnostics, USA*) and transported to the microbiology laboratory of the Aminu Kano Teaching Hospital within 6 h. Samples were inoculated onto MacConckey agar (*Rapid labs UK*) and Salmonella-Shigella agar (*Mast group UK*) and incubated at 37°C for 18–24 h for the primary isolation of enteric bacteria. Colony/colonies were subcultured onto MacConckey agar, and lactose fermenting colonies suspected to be *E. coli* were plated on Eosin Methylene blue agar (EMB) (*Rapid labs UK*) to observe the metallic green sheen, and were then subjected to a battery of conventional biochemical tests (Indole, Citrate, Urease, Methylred, Oxidase, and KIA). Confirmed *E. coli* isolates were preserved in Trypticase soy broth (TSB) (*Biomerieux France*) +20% glycerol.

DNA from a subset of the *E. coli* isolates recovered (215 diarrheic and 33 non- diarrheic individuals) was extracted using standard procedure as described by Dashti et al. (16). Briefly one loop full of *E. coli* from EMB subcultures was suspended in 2 ml of sterile distilled water in an Eppendorf tube, and the bacterial suspension was boiled at 100°C in a water bath for 10 min and centrifuged at 13,000 rpm for 1 min. The supernatant was used as DNA template for a panel of six real-time PCRs for detection of nine VFs (*eae, bfp*A, *elt, est, stx*1, *stx*2, *ehx*A, *agg*R, and *inv*A) as previously described (14). PCRs were run on a thermal cycler CFX 96 (*Bio-Rad*). PCR results were used to classify isolates as typical EPEC (*eae* and *bfp*A positive), atypical EPEC (*eae* positive only), ETEC (*elt* and/or *est* positive), EAEC (*agg*R positive), EIEC (*inv*A positive), EHEC (*stx*1, *stx*2, and *ehx*A positive), and Hybrid (VF typical of more than one pathotype). *E. coli* positive to any VFs were subjected to antimicrobial susceptibility testing using modified Kirby-Bauer disk diffusion method as recommended by CLSI (17) in Mueller-Hinton agar (*Mast group UK*). A panel of 7 antibiotics (*Oxoid UK*) was tested; cefuroxime sodium (CXM), cefotaxime (CTX), amoxicillin clavulanic acid (AMC), ceftazidime (CAZ), ciprofloxacin (CIP), gentamycin (CN), and trimethoprim/sulfamethoxazole (SXT). Results were recorded as susceptible (S, comprising intermediate) or resistant (R) according to the reference zone of inhibition of each antibiotics as per CLSI guidelines (18). Isolates considered resistant to 3 or more classes of antibiotics were classified as multi-drug resistant (MDR). The possible association between social demography and clinical characteristics and the presence of specific VFs was assessed using chi square tests, implemented using SPSS version 20 (*IBM, Colorado, USA*). A *P-*value of < 0.05 was considered indicative of statistically significant differences.

## Results

**Figure 1:**
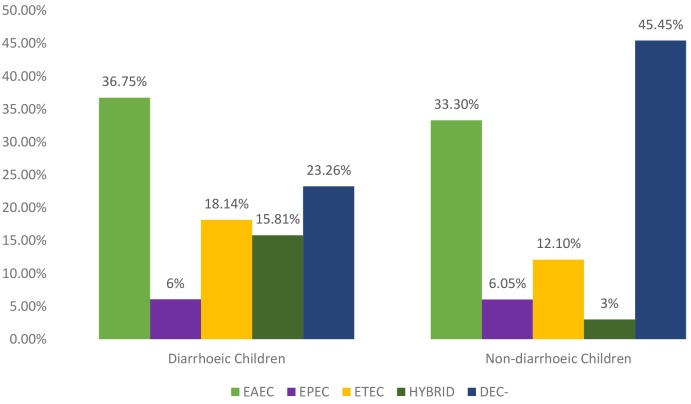
Distribution of DEC pathotypes among cases and control subjects.

The distribution of the VFs in the *E. coli* isolates cultured from the 215 clinical cases (diarrheic) and 33 controls (non- diarrheic) is shown in Table 1. The most prevalent VF in both clinical cases and controls was *agg*R, while no positive results were obtained for *stx*1, *stx*2, and *inv*A. Overall a higher proportion of positive isolates to any VF (isolates in which at least one VF was detected) was found among clinical cases compared to control *E. coli*, although significant (*P* = 0.034) differences were only obtained in the case of *est* (Table 1). The proportion of isolates positive to *agg*R and *est* was significantly different between the three hospitals in the case subjects (*P* < 0.001 and *P* = 0.010 respectively). Based on the VF profile, EAEC was detected in 36.7 and 33.3% of the clinical cases and control subjects, respectively. Hybrid pathotypes were detected in 14.1% of the population (in a significantly higher in the clinical cases, 15.8%, than in the control group, 3.0%, *P* = 0.048) and EAEC/ETEC (8.37%) was the most common combination (Table 2). Samples negative to all VFs (isolates not classified into pathotypes) were found in 45.5% of the controls compared with 23.26% of the clinical cases (Figure 1).

**Table 1:** Distribution pattern of virulence genes detection among case and control under five children in Kano.

**Population**	**Case subjects**				**Control subjects**
**Gene**	**BGH (*N*_**b**_ = 58)** ***n* (%)**	**MMSH (*N*_**m**_ = 71)** ***n* (%)**	**WGH (*N*_**w**_ = 86)** ***n* (%)**	**Total (*N*_**d**_ = 215)** ***n* (%)**	**Control (*N*_**c**_ = 33)** ***n* (%)**
*agg*R +Ve	38 (65.52)	20 (28.17)	52 (60.47)^*^	110 (51.2)	12 (36.36)
*eae* +Ve	11 (18.97)	12 (16.90)	12 (13.95)	35 (16.28)	14 (42.42)
*bfp*A +Ve	2 (3.45)	3 (4.23)	4 (4.65)	9 (4.19)	1 (3.03)
*elt* +Ve	7 (12.07)	15 (21.13)	13 (15.12)	35 (16.28)	3 (9.09)
*est* +Ve	6 (10.34)	20 (28.17)	11 (12.79)^*^	37 (17.21)	1 (3.03)**^#^**
*ehx*A +Ve	0 (0.0)	1 (1.41)	1 (1.16)	2 (0.93)	0 (0.0)

*BGH, Bichi General Hospital; MMSH, Murtala Muhammad Specialist Hospital; WGH, Wudil General Hospital; +Ve, Positive; N_d_, Total Number of Case children; Nc, Total Number of Control children; N_b_, N_m_, and N_w_, Total Number of E. coli screened for Virulence gene in BGH, MMSH, and WGH, respectively*,

* P < 0.05 within diarrheic group,

# *p < 0.05 between diarrheic and non- diarrheic group*.

**Table 2:** Distribution of Diarrheagenic *E. coli* from case and control subjects under 5 years in Kano state.

**Population**	**Case subjects** **(*****N***_****d****_ **=** **215)**				**Control subjects** **(*N*_**n**_ = 33)**	**Total DEC** **(*N* = 248)**
**Pathotype**	**BGH (*N*_**b**_ = 58)** ***N* (%)**	**MMSH (*N*_**m**_ = 71)** ***N* (%)**	**WGH (*N*_**w**__** = **_ 86) *N* (%)**	**Total_**d**_ (*N* = 215)**	**Control (*N*_**c**_ = 33)** ***N* (%)**	
EPEC total	3 (5.17)	**6 (8.45)**	4 (4.65)	13 (6.05)	2 (6.06)	15 (6.05)
Atypical EPEC *eae*	1 (1.7)	4 (5.6)	2 (2.32)	7 (3.26)	1 (3.0)	6 (2.42)
Typical EPEC *bfp*A+*eae*	**2 (3.44)**	2 (2.82)	2 (2.32)	6 (2.79)	1 (3.0)	7 (2.82)
EAEC total	**27 (46.5)**	12 (16.9)	**40 (46.5**)^*^	79 (36.75)	11 (33.3)	90 (36.29)
*agg*R	27 (46.6)	12 (16.9)	40 (46.5)	79 (36.75)	11 (33.3)	
ETEC total	3 (5.17)	**25 (35.2)**	11 (12.8)^*^	39 (18.14)	4 (12.1)	43 (17.34)
*lt* only	1 (1.7)	7 (9.86)	6 (7.0)	14 (6.51)	3 (9.1)	17 (6.85)
*st* only	1 (1.7)	15 (21.13)	3 (3.5)	19 (8.84)	1 (3.0)	20 (8.06)
*st* and *lt*	1 (1.7)	3 (4.23)	2 (2.3)	5 (2.33)	0 (0.0)	6 (2.42)
Hybrid total	**11 (19.0)**	9 (12.7)	14 (16.3)	34 (15.81)	1 (3.0)**^#^**	35 (14.11)
ETEC/EPEC	0 (0.0)	1 (1.4)	3 (3.5)	4 (1.86)	0 (0.0)	4 (1.6)
EPEC/EAEC	5 (8.6)	3 (4.2)	4 (4.7)	12 (5.58)	1 (3.0)	13 (5.24)
EAEC/ETEC	6 (10.34)	5 (7.0)	7 (8.1)	18 (8.37)	0 (0.0)	**18 (7.26)**
Total DEC	44 (75.86)	52 (73.24)	69 (80.23)	165 (76.7)	18 (54.55)	**183 (73.79)**
DEC negative	14 (24.14)	19 (26.76)	17 (19.77)	50 (23.26)	15 (45.45)	65 (26.21)

BGH, Bichi General Hospital; MMSH, Murtala Muhammad Specialist Hospital; WGH, Wudil General Hospital; N_d_, Total Number of Case children; N_n_, Total Number of Control children; N_b_, N_m_, and N_w_, Total Number of E. coli screened for Virulence gene in BGH, MMSH, and WGH, respectively; DEC, Diarrheagenic E. coli, EPEC, Enteropathogenic E. coli; EAEC, Enteroaggregative E. coli Enterotoxigenic E. coli; Total_d_, Total diarrheic;

* P < 0.05 case group,

# *p < 0.05 between case and control group. Numbers in bold indicate higher prevalence*.

The demography and clinical characteristics of subjects with DEC is displayed on Table 3. Detection of DEC pathotypes was more frequent in samples from males (58.2%) than females (41.8%). DEC detection was significantly different depending on age class of the cases (*P* = 0.002): EAEC and EPEC were detected in higher proportions in 0–12 months cases and hybrid detection rate was higher in the >24 months age class. There was a significantly higher detection rate of any of the DEC pathotypes in individuals belonging to the 0–12 months age class in both cases and control subjects (*P* = 0.017). EAEC isolates were detected in a higher proportion among subjects with mixed feeding pattern, EPEC was higher among the “breast feeding only” subjects while ETEC (29.2%) and hybrid pathotypes (29.2%) were higher in those that declared an “artificial only” feeding pattern. There was a significant detection rate of DEC in case subjects (*P* = 0.006) compared to the control subjects. The most common phenotypic resistance in the *E. coli* positive to at least one VF was trimethoprim-sulfamethoxazole (142/183, 77.6%). Overall, there was a higher proportion of resistant isolates among those recovered from the control children compared with those from cases for five antibiotics, although differences were only significant in the case of cefuroxime (50.0% vs. 16.4%; *P* ≤ 0.001), Ceftazidime (66.7% vs. 22.4%; *P* ≤ 0.001), and amoxicillin-clavulanic acid (88.9% vs. 40.0%; *P* ≤ 0.001). Resistance was slightly higher in isolates from clinical cases only in the case of trimethoprim-sulfamethoxazole (78.2% vs. 72.2%) and cefotaxime (12.1% vs. 11.1%) but differences were not significant. The proportion of MDR (mostly resistant to AMC, CIP and SXT) isolates was also higher among those recovered from non- diarrheic children (16.7% vs. 6.1%) (Table 4).

**Table 3:** Distribution of social demography of children with DEC detection among case and control under five children in Kano.

	**Case children (*****N*** **=** **165)**				**Control children (*****N*** **=** **18)**				**Total (*****N*** **=** **183)**	
**Pathotype** **variables**	**EAEC** ***n* = 79**	**EPEC** ***n* = 13**	**ETEC** ***n* = 39**	**Hybrid** ***n* = 34**	**EAEC** ***n* = 11**	**EPEC** ***n* = 2**	**ETEC** ***n* = 4**	**Hybrid** ***n* = 1**	**Total^**d**^** ***n* = 165**	**Total^**n**^** ***n* = 18**
Female	29 (42.0)	5 (7.2)	**23 (33.3)**	12 (17.4)	3 (42.9)	1 (14.3)	2 (28.6)	1 (14.3)	69 (41.8)	7 (38.9)
Male	**50 (52.1)**	**8 (8.3)**	16 (16.7)	**22 (22.9)**	**8 (72.7)**	1 (9.1)	2 (18.2)	0 (0.0)	**96 (58.2)**	11 (61.1)
Age class										
0–12 months	**44 (59.5)**	**9 (12.2)**	13 (17.6)	8 (10.8)^*^	**6 (75.0)**	0 (0.0)	1 (12.5)	**1 (12.5)**	**74 (44.8)**	8 (44.4)**^#^**
13–24 months	20 (33.3)	1 (1.7)	**21 (35.0)**	8 (13.3)	1 (50.0)	0 (0.0)	1 (50.0)	0 (0.0)	60 (36.4)	2 (11.1)
>24 months	15 (48.4)	3 (9.7)	5 (16.1)	**18 (58.1)**	4 (50.0)	**2 (25.0)**	**2 (25.0)**	0 (0.0)	31 (18.8)	8 (44.4)
Feeding										
Artificial	24 (36.9)	3 (4.6)	**19 (29.2)**	**19 (29.2)**	4 (50.0)	2 (25.0)	2 (25.0)	0 (0.0)	65 (39.4)	8 (44.4)
Breast only	10 (52.6)	**3 (15.8)**	3 (15.8)	3 (15.8)	1 (50.0)	0 (0.0)	0 (0.0)	1 (50.0)	19 (11.5)	2 (11.1)
Mixed	**45 (55.6)**	7 (8.6)	17 (21.0)	12 (14.8)	**6 (75.0)**	0 (0.0)	2 (25.0)	0 (0.0)	**81 (49.1)**	8 (44.4)

DEC, Diarrheagenic E. coli; N, Total Number; n, Number obtained; (%), Percentage calculated based on total for each variable; EPEC, Enteropathogenic E. coli; EAEC, Enteroaggregative E. coli Enterotoxigenic E. coli; Total^d^, Total Case; Total^n^, Total Control;

* P < 0.05 within individual group;

# *P < 0.05 between group: numbers in bold indicate higher prevalence*.

**Table 4:** Antibiotic resistance pattern of DEC from case and control children younger than 5 years in Kano.

**Antibiotic**	**EAEC** ***N*** **(%)**		**EPEC** ***N*** **(%)**		**ETEC** ***N*** **(%)**		**Hybrid** ***N*** **(%)**		**Total DEC** ***N*** **(%)**		**DEC Negative**	
	**Case** **(*n* = 79)**	**Control** **(*n* = 11)**	**Case** **(*n* = 13)**	**Control** **(*n* = 2)**	**Case** **(*n* = 39)**	**Control** **(*n* = 4)**	**Case** **(*n* = 34)**	**Control** **(*n* = 1)**	**Case** **(*n* = 165)**	**Control** **(*n* = 18)**	**Case** **(*n* = 50)**	**Control** **(*n* = 15)**
CXM	12 (15.2)	5 (45.5)^*^	2 (15.4)	2 (100.0)	7 (17.9)	1 (25.0)	6 (17.6)	1 (100.0)^*^	27 (16.36)	9 (50.0)^*^	11 (22.0)	6 (40.0)
CTX	10 (12.7)	0 (0.0)	1 (7.7)	0 (0.0)	5 (12.8)	1 (25.0)	4 (11.8)	1 (100.0)^*^	20 (12.12)	2 (11.11)	11 (22.0)	3 (20.0)
CAZ	18 (22.8)	6 (54.5)^*^	2 (15.4)	2 (100.0)	8 (20.5)	3 (75.0)^*^	9 (26.5)	1 (100.0)	37 (22.4)	12 (66.67)^*^	11 (22.0)	10 (66.7)^*^
AMC	25 (31.6)	9 (81.8)^*^	5 (38.5)	2 (100.0)	19 (48.7)	4 (100.0)	17 (50.0)	1 (100.0)	66 (40.0)	16 (88.9)^*^	17 (34.0)	10 (66.7)^*^
CIP	6 (7.6)	1 (9.1)	0 (0.0)	1 (50.0)	1 (2.6)	1 (25.0)^*^	2 (5.9)	0 (0.0)	9 (5.45)	3 (16.67)	9 (18.0)	1 (6.7)
CN	3 (3.8)	0 (0.0)	0 (0.0)	0 (0.0)	1 (2.6)	1 (25.0)^*^	3 (8.8)	0 (0.0)	7 (4.24)	1 (5.56)	5 (10.0)	1 (6.7)
SXT	64 (81.0)	8 (72.7)	8 (61.5)	2 (100.0)	29 (74.4)	2 (50.0)	28 (82.4)	1 (100.0)	129 (78.18)	13 (72.22)	44 (80.0)	6 (40.0)
Res. to 1	67 (84.8)	10 (90.9)	10 (76.9)	2 (100)	34 (87.2)	4 (100.0)	31 (91.2)	1 (100.0)	142 (86.06)	17 (94.44)^*^	47 (94.0)	13 (86.67)
Res. to 2	27 (34.2)	6 (54.5)	0 (0.0)	1 (50.0)	15 (38.5)	1 (25.0)	15 (44.1)	1 (100)	57 (34.55)	9 (50.0)	14 (28.0)	9 (60.0)
MDR	5 (6.3)	1 (9.1)	0 (0.0)	1 (50.0)	2 (5.1)	1 (25.0)	3 (8.8)	0 (0.0)	10 (6.06)	3 (16.67)	7 (14.0)	1 (6.67)

DEC, Diarrheagenic E. coli; N, Number obtained; n, total number of DEC; %, Percentage; EPEC, Enteropathogenic E. coli; EAEC, Enteroaggregative E. coli Enterotoxigenic E. coli; CXM, Cefuroxime; CAZ, Ceftazidime; AMC, Amoxicillin-clavulanic acid; CIP, Ciprofloxacin; CN, Gentamycin; SXT, Trimethoprim-sulfamethoxazole; Res. to 1, 2, Resistant to at least 1 or at least 2 antibiotic classes; MDR, Multidrug Resistance;

* *P < 0.05*.

## Discussion

Diarrhea is one of the most common causes of illness and death in young children in Nigeria (and Kano in particular), but its causative agent is often not determined. In this study we determined the frequency of detection of VFs associated with DEC pathotypes in a subset of isolates recovered from children below 5 years with and without clinical symptoms compatible with a DEC infection in an attempt to establish their clinical significance. To do so, a set of molecular targets for identification of the DEC pathotypes that had already proven their usefulness in the past (9, 14, 19) was used.

Diarrheagenic *E. coli* infections are indistinguishable from gastroenteritis due to other bacterial or viral infection, and therefore isolation and identification of the specific DEC associated with a clinical case could allow caregivers to provide appropriate treatment. The proportion of isolates in which at least one VF specific of relevant DEC pathotypes was found in this study, 76.7 and 54.5% in the diarrheic and non- diarrheic children, respectively, is higher than previous reports in Nigeria of 12.8% (20), with a smaller difference between the two groups compared with previous studies on *E. coli* isolated from rectal swabs of children <5 years in Nigeria (18.4% in case and 2.6% in control) (21). Presence of DEC in clinical patients is not routinely determined in many countries including Nigeria, although advancements in molecular techniques have allowed generating data that may allow a better understanding of the role of DEC as a cause of diarrhea in children. Several studies reported different DEC detection rates in *E. coli* recovered from children <5 years across the globe, ranging between 4 and 87% in Africa [22.9% (22), 7.4% (23), 55.9% (24), 86.5% (25)], Asia [45.2% (26), 4.7% (27), 6.82% (28)], and America 5.5% (29). These differences may be due to the changes in the distribution of DEC pathotypes from region to region and within countries in the same region. This study detected genes for EAEC, EPEC, and ETEC, which is in agreement with other studies performed in Tanzania (22, 30) and Libya (31). No isolates carrying EIEC-specific VFs were found here, similar to what was reported for *E. coli* isolates from children in Peru (32), Tanzania (22, 30), and Libya (31).

The most prevalent VF detected in this study was *agg*R (51.2% in diarrheic and 36.4% in non- diarrheic subjects), which is a transcriptional activator gene required for the expression of the anti-aggregation protein (dispersin) gene *aap* (also called *aspU*) and the antiaggregation protein transporter *aatA* (also known as CVD432 or AA prove) (3). This is one of the most commonly used targets for detection of EAEC (22, 32). The most prevalent pathotype in this study was in fact EAEC (36.8%), which is in agreement with other studies that also described this pathotype as the most frequent in *E. coli* isolates recovered from children with diarrhea (21, 23, 28). Previous studies have demonstrated the importance of EAEC in pediatric cases in developing countries, where it is increasingly recognized as a cause of diarrhea; Ifeanyi et al. (20) in Nigeria, Shah et al. (25) in Kenya, Moyo et al. (22) in Tanzania, Ochoa et al. (32) in Peru, and Tobias et al. (33) in Israel described EAEC as the predominant DEC among children under 5 years. Interestingly, EAEC was the predominant pathotype also in isolates recovered from the control subjects. Previous studies (3) have stated that EAEC is the prevailing pathotype among malnourished children, and therefore its high prevalence in both cases and controls may be therefore attributed to the nutritional status of the children.

ETEC was the second most prevalent pathotype in this study (18.1% in clinical cases and 12.1% in non- diarrheic subjects). A similar pattern (EAEC followed by ETEC as the most prevalent pathotypes) was described previously in Kenya (25) and Libya (31). We detected a higher number of *est*+ ETEC than *elt*+ ETEC, in agreement to the findings of Shah et al. (25), but in contrast with the study of Haghi et al. (34) in Iran, that described a higher proportion of *elt*+ ETEC.

The low proportion of EPEC isolates recovered from samples from clinical cases in this study (6.0%) was also in agreement with a previous study analyzing *E. coli* from fecal samples from children in Egypt (5.2%) (35). The higher proportion of atypical (3.3%) than typical EPEC (2.8%) found in case subjects was also similar to previous findings in children in Iran (2.7% atypical vs. 1% typical) (34). The detection of EPEC-ETEC hybrid pathotypes found here had been already reported in previous studies in Nigeria (20) and India (36), and may be due to the plasticity of *E. coli*, which allows in some cases the combination of VFs from different pathotypes in a single strain (3).

Treating DEC with antibiotics is not routinely recommended; however, understanding the antibiotic susceptibility of these pathogens is important as intestinal *E. coli* strains may serve as a reservoir of antibiotic resistance genes (29). In addition, antimicrobial therapy may be indicated in children with diarrhea due to DEC once identified, and in children with persistent diarrhea. Diarrheagenic *E. coli* may be twice as likely to be resistant to trimethoprim-sulfamethoxazole compared to non-pathogenic isolates (30), which agrees with what was observed in this study: most DEC isolates from both case and control subjects were resistant to trimethoprim-sulfamethoxazole, an antimicrobial commonly used for treating diarrhea and other pediatric diseases in Nigeria. The high resistance levels to trimethoprim-sulfamethoxazole observed here, already described as an emerging problem of DEC isolated from children in other developing countries (37) and for other enteric bacteria worldwide (38), could be therefore the result of its widespread use. In fact, in our study the non-DEC isolates from clinical cases also showed a high resistance level to trimethoprim-sulfamethoxazole (80%, Table 4), what could be a result of the selective pressure favoring resistant isolates due to treatment with this antibiotic.

Even though the proportion of isolates resistant to at least one antimicrobial was high (87%), in agreement with a previous study in children (29), the proportion of MDR isolates (7.1%) was lower compared to previous reports of 24.3% (29) and 32% (39), what could be due to the fact that only 4 classes of antibiotics were used in this study. Globally, EAEC strains have shown high levels of antimicrobial resistance, and our results, reporting a high level of resistance in EAEC isolates to trimethoprim-sulfamethoxazole, are in agreement with previous studies in Africa [Tanzania, 87.5% (30) and Gambia 85% (40)] and South America (76.2%) (32). Similarly, the high levels of resistance to ciprofloxacin in EAEC in this study are similar to reports from Gambia (40). The proportion of EAEC isolates resistant to at least one antibiotic (85.6%) was higher than what was reported in United States (29).

In summary, EAEC was found to be the predominant pathotype among Kano children, and was recovered at higher frequencies from males and patients with 0–12 months. DEC isolates from both cases and controls characterized here were highly resistant to trimethoprim-sulfamethoxazole. Consistent surveillance to determine the prevalence of diarrheal diseases and routine evaluation of diarrheic children for determination of the etiological agent is much needed. Furthermore, additional studies using genotypic and/or phenotypic typing techniques can help to understand the population structure of DEC pathotypes in Kano.

## Data Availability Statement

The datasets generated for this study are available on request to the corresponding author.

## Ethics Statement

The studies involving human participants were reviewed and approved by Kano State Ministry of Health (Operational Research and Advisory Committee). Written informed consent to participate in this study was provided by the participants' legal guardian/next of kin.

## Author Contributions

HS, ND, and BM conceived and design the study. HS and SG-S collected the samples and performed the laboratory analysis, assisted by JA and MU-R. JA and HS analyzed the data and drafted the manuscript. All authors revised the manuscript.

### Conflict of Interest

The authors declare that the research was conducted in the absence of any commercial or financial relationships that could be construed as a potential conflict of interest.
